# Filters of automobile air conditioning systems as in-car source of exposure to infections and toxic moulds

**DOI:** 10.1007/s11356-023-29947-y

**Published:** 2023-09-25

**Authors:** Małgorzata Gołofit-Szymczak, Angelina Wójcik-Fatla, Agata Stobnicka-Kupiec, Rafał L. Górny

**Affiliations:** 1 Department of Chemical, Aerosol and Biological Hazards, Central Institute for Labour Protection—National Research Institute, Warsaw, Poland; 2 Department of Health Biohazards and Parasitology, Institute of Rural Health, Lublin, Poland

**Keywords:** Cars, Air conditioning system, Moulds, Harmful biological agents, Filters, Aflatoxin

## Abstract

The main component of an air conditioning system is air filters. Over time, the filters of an air conditioning system in cars can turn into sources of emission of microbiological hazards. The aim of this study was to quantitatively and qualitatively assess the presence of infectious and toxic fungi in the AC filters in passenger cars. The studied non-woven filters were removed from passenger cars during the “winter”/“summer” seasons. The taxonomic identification of the fungi isolated from the filters was performed using both the culture-based and molecular methods. RT-PCR was applied to assess the presence of gene fragments regulating aflatoxin biosynthesis in the isolates obtained from fungal cultures. The average fungal concentrations in the filter samples collected during the summer/winter season were 5.4 × 10^4^ cfu/m^2^ and 2.4 × 10^4^ cfu/m^2^, respectively. Most of the filter samples, collected in both the studied seasons, revealed the presence of *Aspergillus* species including *A. fumigatus*, *A. niger*, *A. terreus* and/or *A. flavus*. The recorded levels of fungal contamination of AC filters in passenger cars indicate the necessity for more frequent filter replacement in this type of vehicle. Occupational exposure to moulds and the resulting health problems that may be experienced by professional drivers should be properly recognised in order to undertake effective preventive measures.

**Supplementary Information:**

The online version contains supplementary material available at 10.1007/s11356-023-29947-y.

## Introduction

In Europe, the predominant mode of land transportation is by road. Every day, millions of people commute and travel for a few hours using different means of transport, such as private cars, taxis, buses or trucks. The passenger car is the most commonly used means of transport in EU member states. On average, professional drivers spend approximately 8 h/day in their vehicles (Holmer et al. 1995; Jo and Lee 2008; Vonberg et al. 2010).

In order to increase the comfort of travelling, passenger cars are equipped with air conditioning systems. The history of air conditioning dates back to 1930 when the first prototypes of air conditioners were designed in the USA; however, the first fully operating air conditioning system was not installed until 1950. This invention has been considered a milestone in the history of the motor industry, especially in the USA. In Europe, air conditioning systems started to be commonly used in the 1990s and have largely become standard in currently produced cars.

Nowadays, about 90% of new car models are equipped with an air conditioning system (Raţiu et al. 2018). This car equipment option not only contributes to the increased comfort of the driver and passengers, but it is also of vital importance for the safety of driving under various weather conditions (Reyner and Horne 1998). Ventilation and filtration are the two key methods to control the inflow/penetration of ultrafine (UF) and fine particles into the vehicle cabin (Knibbs and Morawska 2012).

Modern air conditioning systems in passenger cars mostly use single-stage filters, made of cellulose, cellulose with non-wovens or artificial fibre, e.g. polyester. Their function is to protect the driver and passengers from biological, industrial and traffic-related air pollutants. The longer the filter is used in such a system, the greater its contamination, due to the absorption of water and accumulation of both organic and inorganic debris which may serve as a source of nutrients (Li et al. 2013). Consequently, over time the air conditioning system (AC) filters can turn into sources of in-car emission of microbiological hazards, including pathogenic mycobiota (Simmons et al. 1999).

When compared with the air conditioning systems applied in dwellings or private houses, the systems used in vehicles seem to be inferior due to the limited space available for their installation. In cars, the small air ducts and frequent changes in air flow direction contribute to the deposition of airborne particles and microorganisms within the air conditioning system (Simmons et al. 1999). Under favourable conditions, biofilm proliferation in the air filter and the air stream passing through it releases health-threatening microbial propagules inside the vehicle cabin, especially if they contain fine fraction particles (< 1.1 μm) (Li et al. 2013).

Numerous scientific reports indicate that both the drivers and passengers in cars and buses can be exposed to hazardous bioaerosols that originate from an air conditioning system (Jo and Lee 2008; Nowakowicz-Dębek et al. 2017; Simmons and Crow 1995; Simmons et al. 1997). The hitherto performed identification analyses have shown that species of the following fungal genera were most commonly isolated: *Penicillium*, *Aspergillus*, *Acremonium*, *Alternaria* and *Cladosporium* (Sattar et al. 2016; Sattar et al. 2017; Simmons et al. 1997). Exposure to moulds, especially via inhalation, may be the cause of different allergic diseases, including conjunctivitis, rhinitis, hay fever, bronchial asthma and allergic alveolitis. In persons with immunodeficiency, severe opportunistic infections may also develop after such exposure (Fiegel et al. 2006; Simmons et al. 1999). The presence and growth of moulds may also result in the release of mycotoxin into the environment. Mycotoxins produced by some of the mould species (including those from the *Aspergillus*, *Penicillium* and *Fusarium* genera) have been found to be toxic to animals and humans (Douwes 2005; Fiegel et al. 2006).

The aim of this study was to quantitatively and qualitatively assess the presence of toxic and infectious fungi in the AC filters in passenger cars, the most common means of transport in EU countries.

## Materials and method

### Filter collection

The analyses were performed using filters removed from the air conditioning system of passenger cars during two measurement periods: “winter season”—a 3-month period from January to March, when the average ambient air temperature was below 10 °C for at least 7 consecutive days, and in “summer season”, slightly longer than the calendar summer—a 5-month period from May to September, when the average ambient air temperature continued to be above 10 °C for at least 7 consecutive days. During each measurement period, new/unused filters were also collected and subjected to further comparative analysis. In each season, the collection of filters was performed from the air conditioning systems of 15 randomly selected passenger cars manufactured between 2019 and 2020.

The tested vehicles were equipped with two types of air conditioning systems:

- Manual—a single-zone air conditioning system is controlled directly by the driver. Interior temperature is regulated by mixing the warm air flow (from the heater) with cold air, but the driver is not able to set the exact temperature inside the vehicle;
- Automatic air conditioning system—this is electronically controlled using readings from sensors placed all over the interior of the car (e.g. external temperature, internal temperature and sun sensors). Depending on the specific system used in the cabin, the supplied air is directed either to the windscreen, the side windows or the leg space. In order to avoid excessive load on the conditioning system, approximately 20% of the air is taken from the outside, the rest deriving from recirculation of the indoor air. The automatic air conditioning systems may be equipped with a multi-segment heater, with a separate control for the driver and the passenger zones (either dual- or four-zone air conditioning systems). Detailed characteristics of the studied vehicles are presented in Table 1.

**Table 1:** Characteristics of the examined passenger cars

Car no.	Year of manufacture	Car mileage (km)	Car mileage since the replacement of cabin air filter (km)	Type of air conditioning system
Winter season measurements				
1/W	2008	165,000	15,000	*Automatic dual-zone*
2/W	2011	37,000	10,000	*Automatic four-zone*
3/W	2016	18,295	6595	Manual
4/W	2009	207,300	20,000	*Automatic dual-zone*
5/W	2011	148,741	120,892	*Automatic dual-zone*
6/W	2009	141,000	40,000	Manual
7/W	2014	37,000	15,000	Manual
8/W	2018	115,000	30,000	*Automatic dual-zone*
9/W	2007	155,000	30,000	*Automatic four-zone*
10/W	2012	158,900	No data available	Manual
11/W	2016	150,204	11,000	Manual
12/W	2015	135,737	119,000	*Automatic dual-zone*
13/W	2007	173,718	162,094	*Automatic four-zone*
14/W	2000	281,021	80,000	*Automatic four-zone*
15/W	2009	148,000	135,000	*Automatic dual-zone*
Summer season measurements				
1/S	2019	41,750	11,750	Manual
2/S	2000	250,000	No data available	Manual
3/S	2013	136,200	15,000	*Automatic four-zone*
4/S	2007	260,000	30,000	*Automatic dual-zone*
5/S	2010	242,500	25,000	Manual
6/S	2011	88,500	15,000	Manual
7/S	2010	289,600	263,500	*Automatic dual-zone*
8/S	2013	133,000	10,000	*Automatic dual-zone*
9/S	2014	201,237	24,737	Manual
10/S	2007	171,000	15,000	*Automatic dual-zone*
11/S	2006	255,600	28,000	*Automatic dual-zone*
12/S	2000	282,688	about 180,000	Manual
13/S	2005	155,000	50,000	*Automatic dual-zone*
14/S	2011	103,600	No data available	*Automatic dual-zone*
15/S	2009	196,400	No data available	*Automatic dual-zone*

### Filter sample processing and analyses

A total of 32 samples from AC filters were separately collected into separate sterile polyethylene bags and subsequently stored in a dry dark place at room temperature (25 °C ± 2) until further analysis.

A square piece of 10 × 10 cm of the filter fabric was cut out using a sterile scalpel, weighed and placed in 100 cm^3^ of 1 g/l of peptone + 5 g/l of NaCl solution. The sample was then shaken on an orbital laboratory shaker for 15 min at 250 rpm and diluted, and then, 100 μl of the resulting suspension was inoculated onto malt extract agar (MEA, Oxoid Ltd., Basingstoke, UK) supplemented with 0.05 g chloramphenicol. After inoculation, agar plates were incubated for 4 days at 30 °C, followed by 4 days at 22 °C. The concentrations of total culturable fungi were expressed as the number of colony-forming units (cfu) on the culture medium per square metre of the examined filter (cfu/m^2^).

### Conventional methodologies

The isolated fungal colonies were directly identified under stereo (model SteREO Discovery V.12, Carl Zeiss, Gottingen, Germany) and light (Nikon) microscopes, based on their macro- and micro-morphological characteristics. In the macroscopic method, fungal isolates were identified on the basis of cultural features of the colony, such as shape, size, pigmentation and type of surface. In the microscopic method, moulds were stained with lactophenol and identified according to their morphology, using several identification keys (Fisher and Cook 1998; Samson et al. 2004; St-Germain and Summerbell 2011). A few yeast colonies were identified by Gram stain and then by the API test (API 20 C AUX, BioMérieux, Marcy l’Etoile, France).

The new/unused filters were also included during each measurement session and analysed in the same way as the filters from the studied cars.

### Molecular methodologies

Species affiliation of the moulds (identified using microscopic methods) was also confirmed with molecular technics.

DNA was isolated from pure fungal cultures grown on MEA plates, using Fungi DNA Mini-Kits (Syngen Biotech, Wrocław, Poland), according to the manufacturer’s protocol. DNA samples were stored at −80 °C until further analysis. The isolated fungal DNA was used as a template in PCR with ITS1 (5′-TCCGTAGGTGAACCTGCGG-3′) and ITS4 (5′-TCCTCCGCTTATTGATATGC-3′) primer sets which allow amplification of the fungal genome fragment located between 18S and 28S rRNA genes, covering the ITS1, 5.8S rRNA and ITS2 fragments. Amplified PCR products were purified, sequenced using a DNA analyser (model 3730, Applied Biosystems, Waltham, USA) and compared with the genotypes from the GenBank database (National Center for Biotechnology Information, US National Library of Medicine, Bethesda, USA) using the BLAST (Basic Local Alignment Search Tool) algorithm (White et al. 1990).

### Molecular detection of selected *Aspergillus* species

In order to confirm the presence of at least 1 of the 4 species of the *Aspergillus* genus, *Aspergillus fumigatus*, *A. niger*, *A. terreus* and/or *A. flavus* in the tested isolates, real-time PCR testing was performed using *Aspergillus* Selective screening kit (Genesig, Chandler’s Ford, UK), with modifications, to increase the sensitivity of the method. An amount of 20 μl of reaction mixture contained 0.5 μl *Aspergillus*_SCRN primer/probe mix (included in the kit), 2 μl of nuclease-free water (included in the kit), 2.5 μl of isolated DNA and 5 μl of iTaq Universal Probes Supermix (Bio-Rad, Hercules, USA). Apart from the control strains, the positive control attached to the *Aspergillus*_SCRN positive control template (FAM) was used. The amplification was performed using the StepOne RT-PCR System (Thermo Scientific, Waltham, USA), under the following conditions: pre-PCR read (holding stage)—30 s at 60 °C, holding stage—2 min at 50 °C and 10 min at 95 °C, followed by 40 cycles: 15 s at 95 °C and 1 min at 60 °C and finally post-PCR read—30 s at 60 °C.

The TaqMan probes used in the experiment were labelled with FAM dye (6-carboxyfluorescein), and fluorescence reading was performed on the blue channel according to manufacturer protocol. The applied tests are characterised by a high sensitivity of ≥ 90, which allows detection from 1 × 10^2^ to 1 × 10^8^ copies of the sought-for genes in the sample. The test result was considered positive when the amplification curve crossed the threshold line, showing the value of the threshold cycle (Cq). The samples with Cq = 28 ± 3, in the absence of amplification in the negative control and with the values of 16 ≤ Cq ≤ 23 for the positive control (as recommended in the protocol), were considered positive. Only the reactions, for which the amplification efficiency was ≥ 90%, were analysed.

### Detection of aflatoxin-producing fungi

The presence of gene fragments regulating the production of aflatoxins in the isolates obtained from fungal cultures was assessed using the PCR method, as described by Scherm et al. (2005). The pairs of primers were used that were complementary to the fragments of structural genes *aflD* and *aflO* or regulatory genes *aflS* and *aflR*, involved in the biosynthesis of aflatoxin B1. The primer sequences are listed in Table 2.

**Table 2:** Primers, target genes, sequences and expected PCR/RT-PCR product sizes in detection of aflatoxin-producing fungi

Primer code	Gene	Primer sequence (5′ → 3′)	PCR product size (bp)	RT-PCR product size (bp)
Nor1-F	*aflD*	5′-ACGGATCACTTAGCCAGCAC-3′	990	812
Nor1-R		5′-CTACCAGGGGAGTTGAGATCC-3′		
OmtB (F)-F	*aflO*	5′-GCCTTGACATGGAAACCATC-3′	1333	1131
OmtB (F)-R		5′-CCAAGATGGCCTGCTCTTTA-3′		
AflJ-F	*aflS*	5′-GAGTCCCTGAGTGTCGGCTA-3′	1450	1004
AflJ-R		5′-TCGGTTGTCATGGTTATCCA-3′		
AflR-F	*aflR*	5′-TCGGTTGTCATGGTTATCCA-3′	999	999
AflJ-R		5′-TCGGTTGTCATGGTTATCCA-3′		

The reaction mix for *aflO*, *aflR* and *aflS* genes, at a total volume of 25 μl, contained 0.2 μl of Taq DNA polymerase (1U, QIAGEN, Germantown, USA), 2.5 μl of 10× PCR buffer containing 15 mM MgCl_2_, 2.5 μl of 2 mM dNTPs (final concentration 0.2 mM), 0.5 μl of 10 μM of each primer pair (final concentration 0.2 μM), 2 μl of isolated DNA and 16.8 μl of nuclease-free water (QIAGEN). The 25 μl of the reaction mixture for the *aflD* gene contained 0.2 μl Taq DNA polymerase (1U; QIAGEN), 2.5 μl 10× PCR buffer containing 15 mM MgCl_2_, 1.5 μl 2 mM dNTPs (final concentration 0.12 mM), 0.25 μl of 10 μM of each primer pair (final concentration 0.1 μM), 2 μl of isolated DNA and 18.3 μl of nuclease-free water (QIAGEN).

PCR testing for the *aflO* gene was performed in a Mastercycler nexus GSX1 thermocycler (Eppendorf, Hamburg, Germany) and for the remaining genes (*aflD*, *aflR* and *aflS*) in the C1000 Thermal Cycler (Bio-Rad), under the following conditions: 5 min at 95 °C (initial denaturation), 35 cycles, each of which included specific denaturation (30 s at 94 °C), primer annealing (60 s at 55 °C), elongation (90 s at 72 °C) and final extension for 7 min at 72 °C. Positive control was the reference strain *Aspergillus flavus* (ATCC-8062), and nuclease-free water (QIAGEN) was used as the negative control.

The detection of amplification products was carried out on 1.5% agarose gels (Prona, Basica LE, Abo Sp. Z O.O., Gdańsk, Poland) and their separation, in a horizontal electrophoresis device (Bio-Rad), with the following parameters: voltage 80 V, current 400 mA and time: 55 min. In order to visualise the results, after electrophoretic separation, the gel was soaked in ethidium bromide solution for 20 min, with a final concentration of 2 μg/cm^3^. The electrophoretic separation image was analysed using an Ingenius Syngene Bio Imaging (Syngene) gel analysis and documentation system. The product was read in the Syngene system in the presence of the following markers: GeneRuler 100 bp (Thermo Scientific) for *aflD* and *aflR* genes (the amplification products were 990 bp and 999 bp, respectively) and DNA Marker Lambda/BstE II (A&A Biotechnology, Gdańsk, Poland) for *aflO* (1333 bp product) and *aflS* (1450 bp product) genes.

### Scanning electron microscope (SEM) imaging (photos enclosed)

In order to illustrate the contamination of air filters with fungi, photos were taken using SEM. A 5 × 5 mm fragment of selected air filters (10 samples) was cut out using a sterile scalpel. Filter surface tests were performed with the use of an SU8010 cold field emission scanning electron microscope (Hitachi, Tokyo, Japan). To improve conductivity, the material was gold-plated using a Q150T ES ion sputter gun (Quorum Technologies, Ltd., Lewes, UK). The observations were carried out at the voltage of 10 kV, WD 10 mm, and at magnifications of × 100, × 500, × 1000 and × 5000.

### Statistical analysis

The data collected during the study were analysed using the Kruskal-Wallis test, Mann-Whitney *U* test, Spearman’s correlation analysis and Fisher tests. The Statistica data analysis software version 7.1. (StatSoft, Inc., Tulsa, USA, 2006) was used. All the relationships with *p* < 0.05 were considered statistically significant.

## Results

### Qualitative and quantitative analyses of filter samples using macro- and microscopic methods

Results of the quantitative analysis of fungal samples collected from non-woven air conditioning system filters during the summer and winter seasons, as well as from new/unused filters, are presented in Table 3.

**Table 3:** Fungal concentrations in the samples collected from non-woven filters (SD—*standard deviation*) (cfu/m^2^)

Fungal concentrations (cfu/m^2^)					
Summer season			Winter season		
Sample	Average value	SD	Sample	Average value	SD
1/S	1.4 × 10^4^	2828.4	1/W	5.3 × 10^3^	353.6
2/S	1.4 × 10^5^	4242.6	2/W	8.5 × 10^3^	707.1
3/S	1.1 × 10^4^	707.1	3/W	8.0 × 10^3^	0
4/S	1.2 × 10^4^	1060.7	4/W	6.8 × 10^3^	1060.7
5/S	4.0 × 10^4^	5303.3	5/W	5.2 × 10^4^	2121.3
6/S	3.0 × 10^4^	4596.2	6/W	1.2 × 10^4^	707.1
7/S	1.8 × 10^5^	26870.1	7/W	9.0 × 10^3^	707.1
8/S	1.8 × 10^4^	4596.2	8/W	9.8 × 10^3^	1767.8
9/S	3.2 × 10^4^	5656.9	9/W	9.3 × 10^3^	353.6
10/S	1.6 × 10^4^	3182.0	10/W	7.2 × 10^3^	2474.9
11/S	1.5 × 10^4^	707.1	11/W	5.8 × 10^4^	3889.1
12/S	1.3 × 10^5^	16970.6	12/W	4.0 × 10^4^	9192.4
13/S	3.5 × 10^4^	3889.1	13/W	5.4 × 10^4^	1060.7
14/S	5.3 × 10^4^	6364.0	14/W	2.8 × 10^4^	12020.8
15/S	8.0 × 10^4^	1767.8	15/W	5.5 × 10^4^	9899.5
Background/S	1.7 × 10^3^	353.6	Background/W	2.2 × 10^3^	353.6

The average fungal concentrations in the filter samples collected during the summer and winter season were 5.4 × 104 cfu/m^2^ and 2.4 × 104 cfu/m^2^, respectively. Comparison of the filters, used with the new/unused, revealed significant differences between them (Kruskal-Wallis test, *p* < 0.01); however, it should be pointed out that even new/unusual filters were already substantially contaminated with fungal conidia.

The comparison of fungal contamination among tested vehicles in both sampling seasons showed statistically significant differences between them (Kruskal-Wallis test, *p* < 0.05). An analysis using Spearman’s correlation coefficient demonstrated that among the tested parameters, the total mileage and the mileage since filter replacement had a significant impact on the level of fungal concentration in non-woven filters (*R* = 0.525 at *p* < 0.003 and *R* = 0.853 at *p* < 0.001, respectively).

The highest fungal concentrations were recorded in the cars marked as 2S, 7S and 12S, with the longest total mileage and the longest mileage since the last air filter replacement during the maintenance of the air conditioning system. In the case of car 2S, the exact mileage since filter replacement was not known, but the odometer readings of 250,000 km indicate a significant use of this vehicle for tasks requiring many thousands of kilometres to be covered.

**Fig. 1:**
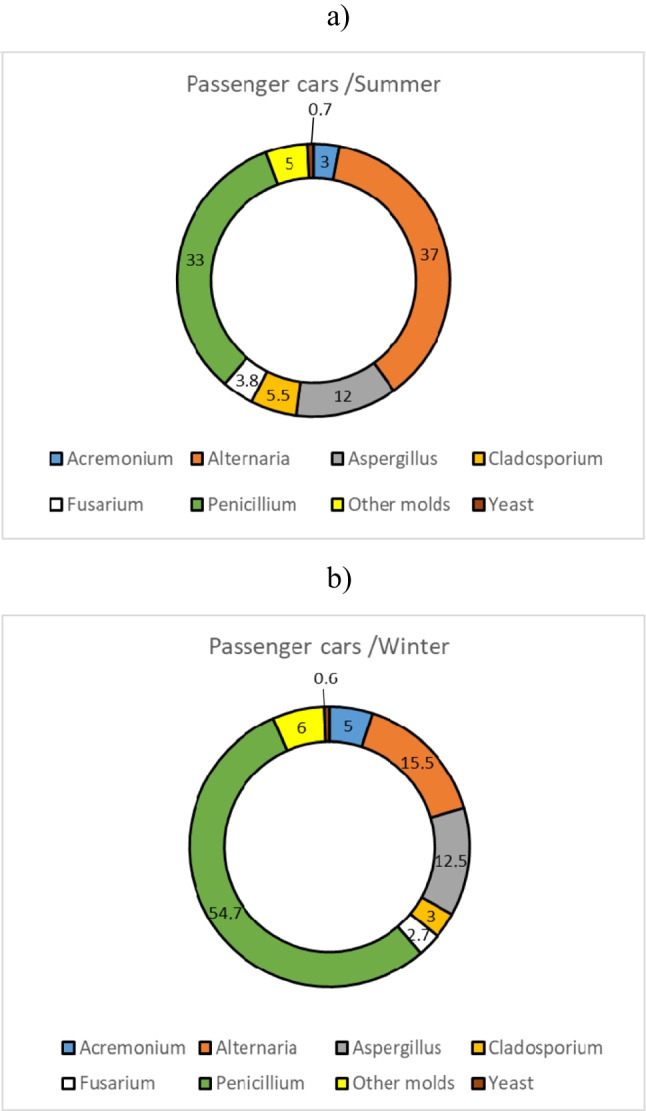
Percentage distributions of microbial groups to the total fungal microbiota isolated from filters in the summer (**a**) and winter seasons (**b**)

Another important parameter in the context of which filter fungal contamination was examined seemed to be the type of air conditioning system (automatic 1, 2, 4-zone or manual). The results showed, however, that this parameter had no significant influence on the fungal concentrations in non-woven filters (*p* > 0.05). The percentage distributions of individual groups of microorganisms in relation to the total fungal microbiota isolated from the filters are shown in Fig. 1.

The *Penicillium* genus was found to prevail (33–54.7%) in the samples from non-woven filters in the vehicles tested in both the summer and winter seasons. The fungi of *Alternaria* (15.7–37%) and *Aspergillus* genera (12–12.5%) were less frequently isolated. All fungal strains isolated from the air filters of the examined cars are listed in Table 4.

**Table 4:** Microorganisms identified in samples from air conditioning system filters

Fungi	Passenger cars/winter	Passenger cars/summer	New/unused filters/winter	New/unused filters/summer
Moulds				
*Acremonium strictum*	x	x		
*Alternaria alternata*	x	x		x
Aspergillus fumigatus**	x	x		
*Aspergillus flavus*	x	x		
*Aspergillus nidulans*	x	x		
*Aspergillus niger*	x	x		
*Curvularia pallescens*		x		
*Cladosporium cladosporioides*	x	x		x
*Dichotomopilus funicola*	x			
*Fusarium culmorum*		x		
*Fusarium solani*	x	x		
*Mucor* spp.		x		
*Penicillium aurantiogriseum*	x	x		
*Penicillium brevicompactum*	x	x	x	x
*Penicillium chrysogenum* Thom		x		
*Penicillium citrinum* Thom	x	x		
*Penicillium citreonigrum*		x		
*Penicillium commune* Thom	x	x	x	x
*Penicillium digitatum*		x		x
*Talaromyces* spp.		x		
*Trichoderma* spp.		x		
*Rhizpopus* spp.	x	x		
*Ulocladium* spp.	x	x		
Yeasts				
*Candida famata*	x	x		
*Candida rugosa*		x		
*Candida ciferrii*		x		
*Rhodotorula minuta*	x	x		

**Pathogenic microorganisms from risk group 2, by risk of infection

**Fig. 2:**
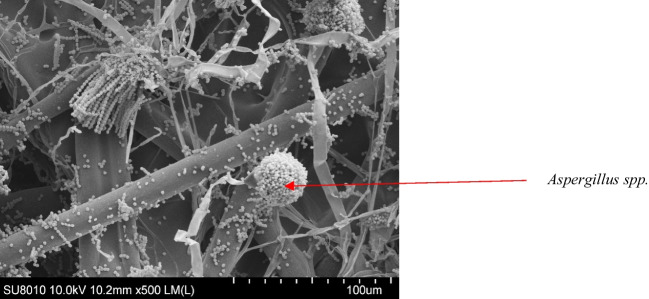
Scanning electron microscopy picture of *Aspergillus* spp. on non-woven air conditioning system filters

From the samples from air filters removed from passenger cars in the summer season, a total of 25 fungal species belonging to 14 genera were isolated. A smaller taxonomic diversity in the fungal microbiota was observed in the winter season. In both seasons, among the most prevalent strains were *Alternaria*, *Aspergillus* and *Penicillium* (Fig. 2). When the species frequency of appearance was analysed, moulds of the *Penicillium* genera (7 species) were most frequently found. Lower species diversity was observed in the new/unused filters in both seasons.

### Molecular identification of selected Aspergillus species

As morphologically similar strains may, in fact, belong to completely different species (Gnat et al. 2017), DNA sequencing is a much more relevant diagnostic approach in mycology allowing precise species identification. In this study, DNA concentrations in the isolates from filter samples ranged from 68.2 to 288.9 ng/μl, while the level of impurities was between 1.6 and 7.0 %.

All the tested filter samples confirmed the presence of ITS1 (5′-TCCGTA GGTGAACCTTGCGG-3′) and ITS 4 (5′-TCCTCC GCTTATTGATATGC-3′) regions, complementary to the highly conserved ribosomal DNA (rDNA) of most fungal species, including the 18S rRNA genes for the small ribosomes and 5.8S rRNA and 28S rRNA for the large ribosomes (Table 2). These genes are present in the cells of all fungi, including yeasts and moulds. The existence of these PCR products indicates the presence of DNA derived from fungal cells. For further molecular analysis in this study, the DNA isolates, for which the ITS1/ITS4 PCR product was obtained, were used.

In order to confirm the presence of at least 1 of 4 species of *Aspergillus* genus, i.e. *Aspergillus fumigatus*, *A. niger*, *A. terreus* and/or *A. flavus* in the tested isolates, RT-PCR tests were performed. The findings for the samples collected during the summer and winter seasons indicated the presence of all *Aspergillus* species: *A. fumigatus*, *A. niger*, *A. terreus* and/or *A. flavus* in the majority of samples (23 of 30—almost 80%). Potentially infectious and toxic mould species were identified in filter samples, regardless of the collection season.

Statistical analysis revealed that such factors as total mileage, type of air conditioning system and the measurement season (summer vs. winter) had no influence on the presence of *Aspergillus fumigatus*, *A. niger*, *A. terreus* and/or *A. flavus* in the filter samples (Fisher’s test, *p* > 0.05).

### Amplification patterns of aflatoxin biosynthesis genes

The RT-PCR test detected transcriptional activation (expression) of *aflS*, *aflD*, *aflR* and *aflO* genes in the samples collected during both studied seasons (Table 5). The DNA sample isolated from filter No. 13S showed amplification of all 4 genes of aflatoxin B1 biosynthesis. In sample No. 15W, amplification of 3 of the sought-for genes—*aflD*, *aflO* and *aflS—*was observed. Statistical analysis showed that the total mileage, the type of air conditioning system and the measurement season did not affect the amplification of *aflS*, *aflD*, *aflR* and *aflO* genes in the filter samples (Fisher’s test, *p* > 0.05).

**Table 5:** Molecular detection (RT-PCR) of the *Aspergillus* genus and expression of gene fragments ITS1 and ITS4, as well as aflatoxin B1 biosynthesis genes in isolates from studied air filters

Sample no.	ITS1/ITS4	*A. fumigatus*, *A. niger*, *A. terreus* or *A. flavus*	*aflD*	*aflO*	*aflS*	*aflR*
1/S	**+**	**+**				
2/S	**+**	**+**				
3/S	**+**	**+**				
4/S	**+**					
5/S	**+**	**+**		**+**		
6/S	**+**		**+**			
7/S	**+**	**+**				
8/S	**+**	**+**				
9/S	**+**	**+**				
10/S	**+**	**+**		**+**		
11/S	**+**	**+**				
12/S	**+**	**+**				**+**
13/S	**+**	**+**	**+**	**+**	**+**	**+**
14/S	**+**	**+**				
15/S	**+**	**+**	**+**			
Background/S	**+**					
1/W	**+**	**+**	**+**			
2/W	**+**	**+**				
3/W	**+**					
4/W	**+**	**+**				
5/W	**+**					
6/W	**+**	**+**				
7/W	**+**	**+**				
8/W	**+**	**+**				
9/W	**+**	**+**	**+**			
10/W	**+**	**+**				
11/W	**+**	**+**	**+**			
12/W	**+**					
13/W	**+**			**+**	**+**	
14/W	**+**		**+**			
15/W	**+**	**+**	**+**	**+**	**+**	
Background/W	**+**					

## Discussion

The findings of this study revealed high concentrations of fungal species in the air conditioning system filters in passenger cars. This refers particularly to the moulds that exhibit infectious and toxic properties.

The air conditioning systems installed in passenger cars are intended to protect the driver and the passengers from exposure to airborne hazards by retaining them on the air filters. However, under favourable conditions, such as long periods of high relative humidity (> 80%), the proliferation of microorganisms on air filters and their subsequent release as bioaerosol inside the vehicle cabin pose a potential health threat, especially when they are a part of the fine airborne fraction. A number of factors, such as high humidity and temperature levels, as well as nutrient availability, contribute to fungal colonisation of the air conditioning devices (Kemp et al. 1995; Pasanen et al. 1993). Many authors have reported that fungal colonies may grow on non-woven filters when relative humidity is high (70–80%) and solid particles as a source of nutrients are present in the air. When nutrient availability for fungi is limited or absent, more intense fungal sporulation may occur, which may result in high numbers of conidia being transmitted from the filter into the air and disseminated within the car cabin (Forthomme et al. 2014; Yang and Johanning 1997). In such situations, the operating air conditioning may enhance the exposure to microbial pathogens (Jo and Lee 2008; Lee and Jo 2005; Li et al. 2013; Sattar et al. 2016; Simmons et al. 1997; Vonberg et al. 2010).

The findings of the present study demonstrate that the air conditioning system filters in passenger cars were contaminated with filamentous fungi and yeasts.

There are no clear guidelines on the life of a cabin filter, often affected by malfunction and irregular replacement. One of the basic criteria for replacing a cabin filter in the process of service inspections of cars is the length of time of its use. Another criterion taken into account when replacing the cabin filter is the number of kilometres travelled during their operation. The conducted research showed that the highest fungal concentrations of 1.8 × 10^5^ cfu/m^2^ of non-woven filter were recorded in the vehicles with the highest mileage (in kilometre) since the last air filter replacement. Similar analyses carried out by other researchers showed that the concentrations of fungi isolated from non-woven filters varied from 5 × 10^2^ to 5 × 10^5^ cfu/m^2^ of the filter (Viegas et al. 2017; Viegas et al. 2018). Viegas et al. (2017), in which the contamination of air conditioning system filters from forklift trucks operating in a waste sorting company, discovered that the fungal colonisation of non-woven filters amounted to 5 × 10^2^ to 5 × 10^5^ cfu/m^2^. In turn, an analysis of microbial contamination of air filters in passenger cars used as taxis showed much lower levels of fungal concentration in filter fabrics, ranging from 5.5 × 10^3^ to 6.8 × 10^4^ cfu/m^2^ (Viegas et al. 2018), which seems to be within the concentration range usually recorded for this type of vehicle.

The duration of using the air condition filter in urban transport vehicles was found to correlate with the fungal colonisation of the filter (Viegas et al. 2018). As reported by Schroder et al. (2017), there is a clear relation between filter age and total airborne fungi and bacteria inside the car space.

A part of the contaminants deposited within the air conditioning system filters may be spread over other parts of the conditioning system, as well as the interior of the car. The impact of the ventilation and air conditioning systems on the modification of bioburden composition in indoor air in buildings or vehicles has also been frequently reported. The reasons for this seem to be related mainly to the lack of preventive maintenance service (Li et al. 2013).

Compared with the air conditioning systems installed in buildings, those used in vehicles are less effective owing to the limited space of the car interior. Aquino et al. (2018) demonstrated that the filters from air conditioning systems in cars provide conditions conducive to the bioaccumulation of several fungal species, including toxic moulds of *Aspergillus* genus which, when released into car cabins, may induce respiratory diseases in the car users.

A comparative analysis of the measurements carried out during the summer and winter seasons revealed that the fungal concentrations in the air filters were significantly higher in the summer season. Seasonal variability is an important factor in modifying the concentration level of microorganisms in the air (Korzeniewska et al. 2008; Korzeniewska et al. 2009). These concentrations ranged from less than 10^2^ cfu/m^3^ in winter months to 10^2^–10^4^ cfu/m^3^ in summertime, which may lead to a higher additive level of filter contamination in the car cabin during the warm season of the year. This confirms the findings from a previous study concerning passenger cars and public transport buses, where the fungal contamination in the passenger area in the summer season was higher than those in the winter season (Giulio et al. 2010; Tseng et al. 2011). Similar conclusions were drawn by Wang et al. who assessed bioaerosol concentrations in train interiors (Wang et al. 2010). In the summer-autumn period, there is also an increase in the number of infections caused by fungi (Buzina et al. 2003). Qualitative analysis indicated that in the filter samples collected during the present study, the most frequently isolated species included moulds of *Penicillium*, *Alternaria* and *Aspergillus* genera. Available scientific literature data point to *Acremonium*, *Aspergillus*, *Alternaria*, *Aureobasidium*, *Cladosporium* and *Penicillium* as the most prevalent species detected in non-woven filters (Aquino et al. 2018; Lee and Jo 2005; Simmons and Crow 1995; Viegas et al. 2018).

In this study, *Aspergillus* section *Fumigati* species *A. fumigatus* and *Aspergillus* section *Flavi* species *A. flavus* were identified during both sampling seasons. The application of the RT-PCR method confirmed the presence of infectious and toxic *Aspergillus* species, section *Fumigati*, *Nigri*, *Terri* and *Flavi* including *A. fumigatus*, *A. niger*, *A. terreus* and/or *A. flavus* among the isolates from filter samples.

The traditional culture-based methods are the most suitable for identifying viable microorganisms that are essential for the assessment of occupational exposure to microbiological hazards and assist in interpreting the level of contamination (Gołofit-Szymczak and Górny 2018). The viable microorganisms, with the capability to grow and reproduce, however, comprise only a small part of the actual microbiota; therefore, focusing only on them may result in the underestimation of human exposure to microbiological threats. Considering that the bioaerosol contains also submicron fragments of microorganic structures, it is estimated that the difference in the concentration of viable and total bioaerosols may be even more pronounced (Gołofit-Szymczak and Górny 2010; Górny et al. 2016). According to Gnat et al. (Gnat et al. 2017), as morphologically similar strains may in fact belong to completely different species, DNA sequencing is a much more suitable diagnostic approach in mycology allowing precise species identification. Taking the above into account, the use of microbial culture analyses, together with molecular testing, allows for a much more accurate identification of the characteristics of microbiota isolated from air filters (Hurley et al. 2019; Viegas et al. 2018).

According to the classification of harmful biological agents included in Directive 2000/54/EC (2000), *A. fumigatus* and *A. flavus* species belong to risk group 2. These microorganisms can cause diseases in humans and can be dangerous to workers but are unlikely to spread to the general population. More than 300 *Aspergillus* species are currently known, including some common human pathogens (Samson et al. 2014). In 2022, the World Health Organisation published a list of fungal priority pathogens with the greatest public health impact and emerging anti-fungal resistance risk. There are 4 pathogens in the critical group, including *Aspergillus fumigatus*.

As reported in the literature, moulds, especially of the *Aspergillus* genera, may pose a particular hazard to human health (Accinelli et al. 2008; Amaike and Keller 2011; Khan et al. 2009; Latgé and Chamilos 2019; Leema et al. 2010; Liu et al. 2018; McCormick et al. 2010). Human infections caused by *Aspergillus* species are considered a major health problem worldwide. *Aspergillus* genus is responsible for more than 80% of pulmonary invasive fungal infections (Segal 2009). *A. fumigatus* and *A. flavus* species are the most common causes of mould-related allergies. These moulds are the source of allergens and may release mycotoxins, volatile organic compounds and glucans into the environment. Fungal allergens are the main cause of atopic diseases, and as many as about 100 fungal species are linked to the symptoms of allergic respiratory diseases. This refers mostly to *Penicillium notatum*, *Aspergillus fumigatus* and *Cladosporium herbarum* (Amaike and Keller 2011; Kurup and Banerjee 2000; Latgé and Chamilos 2019; McCormick et al. 2010). The allergic respiratory diseases related to moulds include, among others, bronchial asthma, allergic alveolitis, organic dust toxic syndrome (ODTS), byssinosis and chronic bronchial inflammation.

Among different moulds, *A. fumigatus* and *A. flavus* can relatively easily induce opportunistic infections in healthy persons with weakened immune functions. Such exposure may also lead to severe respiratory illnesses such as bronchial asthma or mucoviscidosis (Amaike and Keller 2011; McCormick et al. 2010). They have been also recognised as the cause of invasive aspergillosis in immunodeficient patients (Latgé and Chamilos 2019; Leema et al. 2010).

Effective treatment of fungal infections depends on the proper selection of the anti-fungal agents. Anti-fungal resistance limits current treatment strategies. Azoles are the first-line drugs used to treat diseases caused by *A. fumigatus.* Azoles are the only group of compounds used both in medicine and in agriculture as plant protection products. There are 2 routes for *A. fumigatus* isolates to acquire resistance to azoles: (1) the clinical route, associated with the use of triazoles in medicine to treat patients suffering from fungal infections, and (2) the environmental route, mainly related to the use of azoles in agriculture to protect plants against fungal pathogens. Such a widespread use of azoles leads to the selection of resistant strains (Rivelli Zea and Toyotome 2022; Sanglard 2016; Wei et al. 2015).

Several clinical and field studies have been conducted on the expression of aflatoxin biosynthesis genes in the isolates of *A. flavus* (Accinelli et al. 2008; Barakat et al. 2019; Kelly et al. 1997; Leema et al. 2010). The current study is possibly the first to investigate the expression of regulatory and structural genes of aflatoxin biosynthesis in *A. flavus* isolates from air conditioning system filters in passenger cars. The amplification of 4 gene fragments (*aflS*, *aflD*, *aflR* and *aflO*), encoding the proteins involved in the biosynthesis of B1 aflatoxin, revealed the presence of specific PCR products in 40% of tested cars. The presence of these genes in the tested material indicates the existence of the fungi potentially capable of aflatoxin B1 biosynthesis.

The findings of many studies indicate that the RT-PCR method may be highly effective for the differential analysis of aflatoxin-produced strains of *A. flavus*, as it enables the detection of *aflS*, *aflD*, *aflR* and *aflO* transcripts (Chang 2003; Ehrlich et al. 2003). Identification of the genes that are essential in the aflatoxin biosynthesis pathway, along with the assessment of their expression, may be used as a marker of the aflatoxigenic potential of *A. flavus* (Scherm et al. 2005).

Aflatoxins are produced by the toxigenic strains of *Aspergillus* species, mainly *A. flavus* and *A. parasiticus.* Among aflatoxins, aflatoxin B1, B2, G1 and G2 exhibit the strongest biological effect. Aflatoxin B1 is transformed in lung epithelial cells by cytochrome p450 into an active metabolite (exo-AFB1-8,9-epoxide) capable of reacting with DNA, which indicates its potential carcinogenic properties. Aflatoxins have been recognised to be among the strongest mutagens, teratogens and carcinogens (Benkerroum 2020; Henry et al. 2002; Montesano et al. 1997). The International Agency for Research on Cancer has classified all aflatoxins as group 1 human carcinogens (2012). Aflatoxins derive from a part of the strains of *Aspergillus* species only, and the proportion of toxigenic strains, as well as the concentration of mycotoxins they produce, depends, among others, on soil and microclimatic conditions. Scientific literature reports show that for *A. flavus*, the percentage of its toxigenic strains ranges from 20 to 98%, depending on the medium from which they were isolated (Benkerroum 2020; Bilgrami and Choudhary 1993).

This study confirms (which seems to apply also to occupational settings) that the assessment of health risk from exposure to mycobiota deposited in and released from air conditioning system filters should additionally take into account exposure not only to the bioburden itself but to its metabolites (e.g. mycotoxins).

## Conclusions

- The air conditioning systems installed in passenger cars provide suitable conditions for infectious and toxigenic fungal species to proliferate. With the increasing duration of air filter use, the contaminated filter may become a source of fungal emission that poses health risks to the driver and passengers.
- Among the detected mould species, *Aspergillus flavus* and *Aspergillus fumigatus* were identified which have been classified (Directive 2000/54/EC) as pathogens from risk group 2 and additionally labelled as having potential allergic effects. The presence of these species at high concentrations is considered to be a serious health hazard to the exposed individuals.
- The use of highly sensitive and specific molecular methods, coupled with traditional culture-based methods, allows for a much more accurate identification of mycobiota isolated from air conditioning system filters and, by that, for a very precise assessment of exposure to microbiological hazards.
- In light of the above, air conditioning system filters seem to be a useful tool that may help evaluate the driver’s and passengers’ exposure to biologically active microbial propagules.
- The recorded levels of microbiological contamination of non-woven air filters in passenger cars indicate the necessity for more frequent filter replacement in these types of vehicles. It is recommended to replace them at least once a year or after 15,000 km. This may prevent the transmission of bioaerosols inside the cabin and the potential in-car exposure to airborne microbiological hazards.

### Supplementary information

ESM 1 (DOCX 14 kb)

ESM 2 (PDF 513 kb)

## Data Availability

Not applicable.
