# Supplementary material for: Filters of automobile air conditioning systems as in-car source of exposure to infections and toxic moulds
Source: Environ Sci Pollut Res Int. 2023 Sep 25;30(49):108188–200. doi: 10.1007/s11356-023-29947-y (PMC10611836; doi:10.1007/s11356-023-29947-y)
Supplement: Supplementary file 1 — (DOCX 14 kb) [file 11356_2023_29947_MOESM1_ESM.docx]

Małgorzata Gołofit-Szymczak^1^, Angelina Wójcik-Fatla^2^, Agata Stobnicka-Kupiec^1^, Rafał L. Górny^1^

^1^Department of Chemical, Aerosol and Biological Hazards, Central Institute for Labour Protection – National Research Institute, Warsaw, Poland

^2^Department of Health Biohazards and Parasitology, Institute of Rural Health, Lublin, Poland

Corresponding author:

Małgorzata Gołofit-Szymczak

magol@ciop.pl, phone (+48) 22 623 46 82; fax (+48) 22 623 36 93

Department of Chemical, Aerosol and Biological Hazards Central Institute for Labour Protection – National Research Institute, Warsaw, Poland

**Filters of automobile air conditioning systems as in - car source of exposure to infections and toxic moulds**

**Molecular detection of selected *Aspergillus* species**

In order to confirm the presence of at least 1 of the 4 species of the *Aspergillus* genus: *Aspergillus fumigatus*, *A. niger*, *A. terreus* and/or *A. flavus* in the tested isolates, real-time PCR testing was performed using *Aspergillus* - Selective screening kit (Genesig, Chandler's Ford, UK), with modifications, to increase the sensitivity of the method. An amount of 20 µl of reaction mixture contained 0.5 µl *Aspergillus*_SCRN primer/probe mix (included in the kit), 2 µl of nuclease-free water (included in the kit), 2.5 µl of isolated DNA, and 5 µl of iTaq Universal Probes Supermix (Bio-Rad, Hercules, USA). Apart from the control strains, the positive control attached to the *Aspergillus*_SCRN positive control template (FAM) was used. The amplification was performed using the StepOne RT-PCR System (Thermo Scientific, Waltham, USA), under the following conditions: Pre-PCR Read (holding stage) - 30 seconds at 60°C, holding stage - 2 minutes at 50°C and 10 minutes at 95°C, followed by 40 cycles: 15 seconds at 95°C and 1 minute at 60°C, and finally Post PCR-Read - 30 seconds at 60°C.

The TaqMan probes used in the experiment were labelled with FAM dye (6-carboxylfluorescein), and fluorescence reading was performed on the blue channel according to manufacturer protocol. The applied tests are characterized by a high sensitivity of ≥ 90, which allows detection from 1x10^2^ to 1x10^8^ copies of the sought-for genes in the sample. The test result was considered positive when the amplification curve crossed the threshold line, showing the value of the threshold cycle (Cq). The samples with Cq = 28 ± 3, in the absence of amplification in the negative control and with the values ​​of 16 ≤ Cq ≤ 23 for the positive control (as recommended in the protocol), were considered positive. Only the reactions, for which the amplification efficiency was ≥ 90%, were analysed.

Table 1. Samples name

| Sample No. on *Aspergillus* - Selective screening kit | Filter sample name |
| --- | --- |
| A3 | 1/S |
| A4 | 2/S |
| A5 | 3/S |
| A6 | 4/S |
| A7 | 5/S |
| A8 | 6/S |
| B1 | 7/S |
| B2 | 8/S |
| B3 | 9/S |
| B4 | 10/S |
| B5 | 11/S |
| B6 | 12/S |
| B7 | 13/S |
| B8 | 14/S |
| C1 | 15/S |
| E5 | Background/S |
| F5 | 1/W |
| F4 | 2/W |
| D5 | 3/W |
| C2 | 4/W |
| C3 | 5/W |
| C4 | 6/W |
| C5 | 7/W |
| C6 | 8/W |
| C7 | 9/W |
| C8 | 10/W |
| D4 | 11/W |
| D5 | 12/W |
| D6 | 13/W |
| D7 | 14/W |
| D8 | 15/W |
| E5 | Background/W |
