# Supplementary material for: Filters of automobile air conditioning systems as in-car source of exposure to infections and toxic moulds
Source: Environ Sci Pollut Res Int. 2023 Sep 25;30(49):108188–200. doi: 10.1007/s11356-023-29947-y (PMC10611836; doi:10.1007/s11356-023-29947-y)
Supplement: Supplementary file 2 — (PDF 513 kb) [file 11356_2023_29947_MOESM2_ESM.pdf]

Experiment Results Report

awf+aspergillus_wersja3

Experiment Summary

**Experiment Name**	:awf+aspergillus_wersja3
**Experiment Type**	:Presence/Absence
**File Name**	:awf+aspergillus_wersja3.eds
**Run Started**	:2021 Aug 02 4:21:51 PM
**Run Finished**	:2021 Aug 02 5:56:36 PM
**Run Duration**	:94 minutes 44 seconds
**Date Modified**	:2021 Aug 02 4:21:28 PM
**User**	:
**Number of wells used**	:48
**Number of wells with results**	:48
**Instrument Name**	:Local Instrument
**Instrument Type**	:Applied Biosystems StepOne™ Instrument
**Comments**	:

Plate Layout

	1	2	3	4	5	6	7	8
A	neg1<br>N Target 1<br>FAM-NFQ-MGB	neg2<br>N Target 1<br>FAM-NFQ-MGB	1<br>U Target 1<br>FAM-NFQ-MGB	2<br>U Target 1<br>FAM-NFQ-MGB	3<br>U Target 1<br>FAM-NFQ-MGB	4<br>U Target 1<br>FAM-NFQ-MGB	5<br>U Target 1<br>FAM-NFQ-MGB	6<br>U Target 1<br>FAM-NFQ-MGB
B	7<br>U Target 1<br>FAM-NFQ-MGB	8<br>U Target 1<br>FAM-NFQ-MGB	9<br>U Target 1<br>FAM-NFQ-MGB	10<br>U Target 1<br>FAM-NFQ-MGB	11<br>U Target 1<br>FAM-NFQ-MGB	12<br>U Target 1<br>FAM-NFQ-MGB	24<br>U Target 1<br>FAM-NFQ-MGB	23<br>U Target 1<br>FAM-NFQ-MGB
C	22<br>U Target 1<br>FAM-NFQ-MGB	21<br>U Target 1<br>FAM-NFQ-MGB	20<br>U Target 1<br>FAM-NFQ-MGB	19<br>U Target 1<br>FAM-NFQ-MGB	14<br>U Target 1<br>FAM-NFQ-MGB	15<br>U Target 1<br>FAM-NFQ-MGB	16<br>U Target 1<br>FAM-NFQ-MGB	17<br>U Target 1<br>FAM-NFQ-MGB
D	18<br>U Target 1<br>FAM-NFQ-MGB	25<br>U Target 1<br>FAM-NFQ-MGB	26<br>U Target 1<br>FAM-NFQ-MGB	27<br>U Target 1<br>FAM-NFQ-MGB	28<br>U Target 1<br>FAM-NFQ-MGB	29<br>U Target 1<br>FAM-NFQ-MGB	30<br>U Target 1<br>FAM-NFQ-MGB	31<br>U Target 1<br>FAM-NFQ-MGB
E	13<br>U Target 1<br>FAM-NFQ-MGB	32<br>U Target 1<br>FAM-NFQ-MGB	33<br>U Target 1<br>FAM-NFQ-MGB	34<br>U Target 1<br>FAM-NFQ-MGB	35<br>U Target 1<br>FAM-NFQ-MGB	36<br>U Target 1<br>FAM-NFQ-MGB	48<br>U Target 1<br>FAM-NFQ-MGB	47<br>U Target 1<br>FAM-NFQ-MGB
F	46<br>U Target 1<br>FAM-NFQ-MGB	45<br>U Target 1<br>FAM-NFQ-MGB	44<br>U Target 1<br>FAM-NFQ-MGB	43<br>U Target 1<br>FAM-NFQ-MGB	42<br>U Target 1<br>FAM-NFQ-MGB	asp<br>U Target 1<br>FAM-NFQ-MGB	asp2<br>U Target 1<br>FAM-NFQ-MGB	aspujemn<br>U Target 1<br>FAM-NFQ-MGB

Presence/Absence Plot

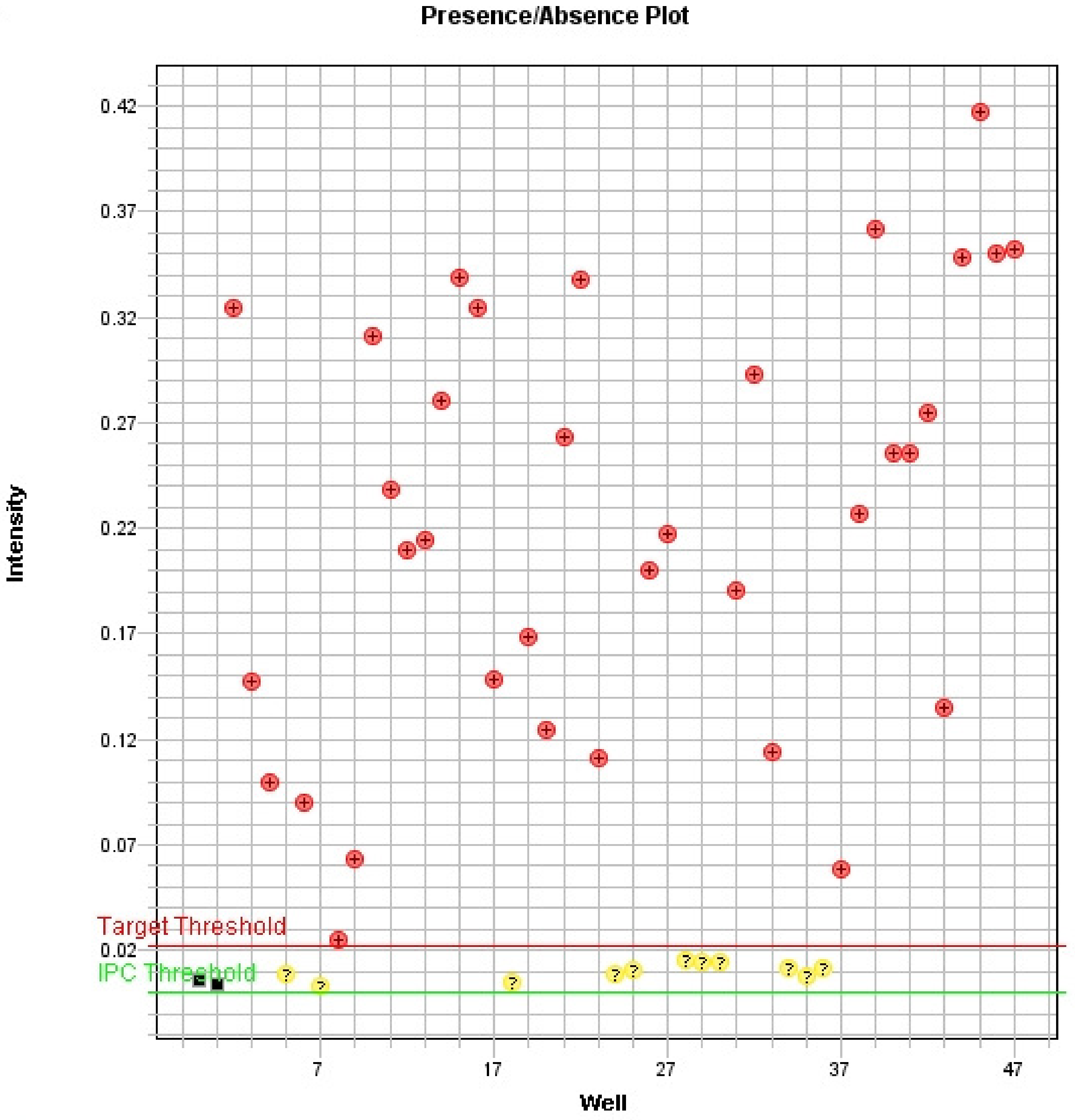

Legend

Unknown Calls:	Presence	Absence	Unconfirmed
IPC Calls:	IPC Failed	IPC Succeeded	
Controls:	Negative Control	Blocked IPC Control	

Amplification Plot (ΔRn vs. Cycle)

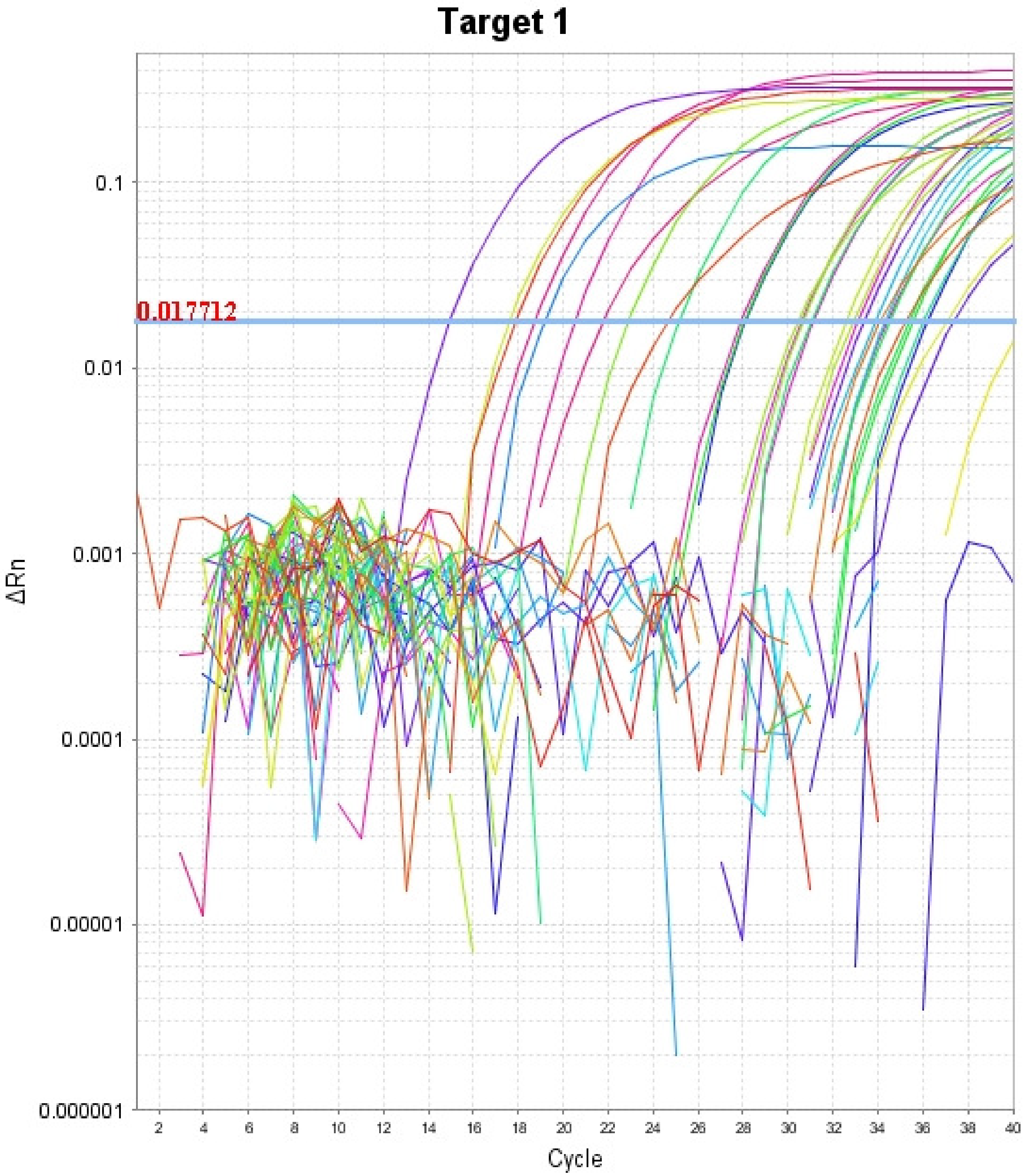

Results Table

Well	Sample	#Flags	Target	Task	Dye	Threshold	ΔRn	Call
A1	neg1	0	Target 1	NTC	FAM-NFQ-		0.0061	Negative Control
A2	neg2	0	Target 1	NTC	FAM-NFQ-		0.0042	Negative Control
A3	1	0	Target 1	UNKNOWN	FAM-NFQ-	0.0224	0.3245	Presence
A4	2	0	Target 1	UNKNOWN	FAM-NFQ-	0.0224	0.1478	Presence
A5	3	0	Target 1	UNKNOWN	FAM-NFQ-	0.0224	0.0993	Presence
A6	4	0	Target 1	UNKNOWN	FAM-NFQ-	0.0224	0.0091	Unconfirmed
A7	5	0	Target 1	UNKNOWN	FAM-NFQ-	0.0224	0.0897	Presence
A8	6	0	Target 1	UNKNOWN	FAM-NFQ-	0.0224	0.0029	Unconfirmed
B1	7	0	Target 1	UNKNOWN	FAM-NFQ-	0.0224	0.0252	Presence
B2	8	0	Target 1	UNKNOWN	FAM-NFQ-	0.0224	0.063	Presence
B3	9	0	Target 1	UNKNOWN	FAM-NFQ-	0.0224	0.3113	Presence
B4	10	0	Target 1	UNKNOWN	FAM-NFQ-	0.0224	0.2386	Presence
B5	11	0	Target 1	UNKNOWN	FAM-NFQ-	0.0224	0.2098	Presence
B6	12	0	Target 1	UNKNOWN	FAM-NFQ-	0.0224	0.2141	Presence
B7	24	0	Target 1	UNKNOWN	FAM-NFQ-	0.0224	0.2808	Presence
B8	23	0	Target 1	UNKNOWN	FAM-NFQ-	0.0224	0.3386	Presence
C1	22	0	Target 1	UNKNOWN	FAM-NFQ-	0.0224	0.3246	Presence
C2	21	0	Target 1	UNKNOWN	FAM-NFQ-	0.0224	0.1483	Presence
C3	20	0	Target 1	UNKNOWN	FAM-NFQ-	0.0224	0.0046	Unconfirmed
C4	19	0	Target 1	UNKNOWN	FAM-NFQ-	0.0224	0.1686	Presence

Well	Sample	#Flags	Target	Task	Dye	Threshold	ΔRn	Call
C5	14	0	Target 1	UNKNOWN	FAM-NFQ-	0.0224	0.125	Presence
C6	15	0	Target 1	UNKNOWN	FAM-NFQ-	0.0224	0.2637	Presence
C7	16	0	Target 1	UNKNOWN	FAM-NFQ-	0.0224	0.3379	Presence
C8	17	0	Target 1	UNKNOWN	FAM-NFQ-	0.0224	0.1113	Presence
D1	18	0	Target 1	UNKNOWN	FAM-NFQ-	0.0224	0.0089	Unconfirmed
D2	25	0	Target 1	UNKNOWN	FAM-NFQ-	0.0224	0.0107	Unconfirmed
D3	26	0	Target 1	UNKNOWN	FAM-NFQ-	0.0224	0.2006	Presence
D4	27	0	Target 1	UNKNOWN	FAM-NFQ-	0.0224	0.2169	Presence
D5	28	0	Target 1	UNKNOWN	FAM-NFQ-	0.0224	0.0158	Unconfirmed
D6	29	0	Target 1	UNKNOWN	FAM-NFQ-	0.0224	0.015	Unconfirmed
D7	30	0	Target 1	UNKNOWN	FAM-NFQ-	0.0224	0.0145	Unconfirmed
D8	31	0	Target 1	UNKNOWN	FAM-NFQ-	0.0224	0.1909	Presence
E1	13	0	Target 1	UNKNOWN	FAM-NFQ-	0.0224	0.2929	Presence
E2	32	0	Target 1	UNKNOWN	FAM-NFQ-	0.0224	0.1144	Presence
E3	33	0	Target 1	UNKNOWN	FAM-NFQ-	0.0224	0.012	Unconfirmed
E4	34	0	Target 1	UNKNOWN	FAM-NFQ-	0.0224	0.0075	Unconfirmed
E5	35	0	Target 1	UNKNOWN	FAM-NFQ-	0.0224	0.0114	Unconfirmed
E6	36	0	Target 1	UNKNOWN	FAM-NFQ-	0.0224	0.0582	Presence
E7	48	0	Target 1	UNKNOWN	FAM-NFQ-	0.0224	0.2271	Presence
E8	47	0	Target 1	UNKNOWN	FAM-NFQ-	0.0224	0.3616	Presence
F1	46	0	Target 1	UNKNOWN	FAM-NFQ-	0.0224	0.2553	Presence

Well	Sample	#Flags	Target	Task	Dye	Threshold	ΔRn	Call
F2	45	0	Target 1	UNKNOWN	FAM-NFQ-	0.0224	0.2552	Presence
F3	44	0	Target 1	UNKNOWN	FAM-NFQ-	0.0224	0.2746	Presence
F4	43	0	Target 1	UNKNOWN	FAM-NFQ-	0.0224	0.1351	Presence
F5	42	0	Target 1	UNKNOWN	FAM-NFQ-	0.0224	0.3488	Presence
F6	asp	0	Target 1	UNKNOWN	FAM-NFQ-	0.0224	0.4175	Presence
F7	asp2	0	Target 1	UNKNOWN	FAM-NFQ-	0.0224	0.3505	Presence
F8	aspujemn	0	Target 1	UNKNOWN	FAM-NFQ-	0.0224	0.3523	Presence

QC Summary

Total Wells	48	Processed Wells	48	Targets Used	1
Well Setup	48	Flagged Wells	0	Samples Used	48

Flag	Name	Frequency	Locations
AMPNC	Amplification in negative control	0	
BADROX	Bad passive reference signal	0	
BLFAIL	Baseline algorithm failed	0	
CTFAIL	Cт algorithm failed	0	
EXPFAIL	Exponential algorithm failed	0	
NOAMP	No amplification	0	
NOISE	Noise higher than others in plate	0	
NOSIGNAL	No signal in well	0	
OFFSCALE	Fluorescence is offscale	0	
SPIKE	Noise spikes	0	
THOLDFAIL	Thresholding algorithm failed	0	
